# Diagnostic Challenges of High-Functioning Autism Spectrum Disorder in Females

**DOI:** 10.7759/cureus.13006

**Published:** 2021-01-30

**Authors:** Mayank Gupta, Ridhima Chaudhary

**Affiliations:** 1 Psychiatry, Lake Erie College of Osteopathic Medicine, Erie, USA; 2 Psychiatry and Behavioral Sciences, Clarion Psychiatric Center, Clarion, USA; 3 Psychiatry and Behavioral Sciences, Santokba Durlabhji Hospital, Jaipur, IND

**Keywords:** female, autism

## Abstract

In the last decade, research about distinct female phenotypes in autism spectrum disorders has gained momentum. These patients are often undiagnosed since they do not meet the diagnostic criterion. And since the current standardized instruments are based on the same criterion, these assessments may often lead to false negative results. In clinical settings these patients have a distinct presentation, challenges, and impairments. These findings are useful in understanding, early identification, and effectively treating these individuals. We present a case series to highlight these issues and summarize the recent advances in research.

## Introduction

Asperger’s syndrome (AS) was first described by Hans Asperger in 1944. But it was Lorna Wing in 1981 who formally presented it as a distinct condition separate from autism [1]. After a decade of research, it was included in Diagnostic and Statistical Manual of Mental Disorders (DSM IV). Since its inclusion in DSM IV (American Psychiatric Association, 1994), there has been constant debate on whether it is a part of ASD or a distinct entity by itself. In late 1990s as more research emerged, the term high-functioning autism (HFA) was used interchangeably with AS [2]. Several studies have rejected that AS has different developmental trajectory compared to ASD [3]. Therefore, in 2013 in DSM V, AS was excluded due to lack of evidence that they are different. Since then there has been literature that raises questions on whether removal of AS from DSM V was premature [4-5]. According to the Center for Disease Control (CDC), approximately one in 54 children in the United States is diagnosed with an ASD. And among them, one in 34 are boys and one in 144 are girls. Interestingly, in 2020, CDC has released new study data estimating about 2.21% adults in the United States have ASD, and among them 3.62% are male adults and 0.86% are female adults.

In clinical practice, patients with AS are diagnosed late in life and often struggle with poor social outcomes and quality of life [6]. They underperform occupationally and have been socially excluded. Females with high-functioning ASD are diagnosed even later in life as compared to males. These females initially present with behavioral, affective, and anxiety symptoms. In many instances, they have multiple previous diagnosis and do not show progress or improvement with several evidence-based interventions. These females lack distinct profile of ASD and therefore lead to unique challenges in dealing with this condition. Patient and caregivers have difficulty accepting alternative explanations due to lack of robust evidence in clinical practice. These issues further delay diagnosis and intervention. Such females are an under-represented subgroup among ASD with atypical presentation. However, in the last few years there has been more research to identify these individuals who have difficulty meeting the criterion even though they have significant impairment. Camouflaging is a broad construct seen more often in females and is described as masking of ASD symptoms with motivation to assimilate [7]. The consequence of camouflaging is mental exhaustion and subsequently heightened anxiety. Compensation is slightly different from camouflaging and is described as enhancing tendencies to compensate for these deficits [8]. The conventional understanding about deficits in the theory of mind tasks that lead to social difficulties was challenged in these new researches. These very individuals with enhanced social skills and poor performance in the theory of mind tasks are the ones who are difficult to meet the diagnostic criterion. It has been of interest to understand this enigma with recent advances in research. We would therefore like to present four cases of females with HF-ASD.

## Case presentation

Case I

Patient is an 11-year-old female sixth-grade student. She was referred to the psychiatric clinic by her pediatrician for mood swings, irritability, and stubbornness. Parents were concerned about her school refusal. Mother reported that she refuses to follow instructions, and these symptoms are getting worse. She was afraid of visiting hospitals, and during her initial visit she had difficulty in engaging and had minimal interaction with the clinicians. During her assessment, the mother reported that she was born preterm at eight months of gestation with immediate birth cry. The patient had no developmental delays, and the speech was acquired at 1.3 years of age. The patient had deep interest in mathematics, science, and technology but did not like going to school. She did not like to interact with kids of her age. She struggled to make friends, engaging in reciprocal play, and going to a new place. She refused to go on the school bus with other children. She had a deep knowledge about dogs and their breeds. She liked playing with building blocks and watched the same TV series multiple times. She was previously diagnosed with obsessive compulsive disorder (OCD) and anxiety disorder and received treatment from two different psychiatrists. There was no relevant medical history. During assessment she was shy and attempted to make eye contact but could not sustain it. She had sensory issues like sensitivity toward rough textured clothing, strong smell, and aversion toward loud noises. She did not wear undergarments till three years of age and avoided haircuts. IQ assessment was conducted with some difficulty since it was challenging to develop a rapport. Wechsler Intelligence Scale for Children - Fourth Edition (WISC-IV) was conducted for IQ testing, which revealed a scattered profile with full-scale IQ in average intellectual functioning range with low processing speed. She scored 38 on childhood autism rating scale high functioning (CARS2-HF), signifying severe symptoms of autism spectrum disorder. The patient was initiated on aripiprazole 2 mg once daily for irritability and started behavioral therapy three times a week. The parents were provided psychoeducation about her diagnosis and treatment strategies. At four weeks follow-up visit, the family reported reduction in irritability and improvement in overall behaviors and academic performance.

Case II

Patient is a 22-year-old female who was referred for psychiatric consultation by her therapist for the symptoms of low mood, poor motivation, and anhedonia. She reported these symptoms have been present for the last one year but has been progressively getting worse. She started therapy a month ago but missed several appointments. She also had difficulties at work for not being able to complete her assignments. Her initial complaints were that she was not able to understand self, often gets bored when among people, and at the same time fearful of being alone. During assessment, she reported about deep interest in quantum physics as early as seventh grade. She was a honor roll student throughout her academic career and has won prestigious awards for physics at the national level. However, she struggled to maintain friendships and also stated that on many occasions she felt no need to have friends. In high school she was the victim of bullying due to the way she dressed and failed to maintain romantic relationships. She experienced emotional and sexual abuse by her boyfriend at the age of 19. She was uncomfortable with the development of secondary sexual characters during late adolescence and felt difficult to fit in with her peers. She liked reading science books and learning German language as a coping strategy to deal with stressful situations. She also had one previous trial on antidepressant medication (sertraline 50 mg daily) for two months without any benefit. After the consultation, we discussed the possibility of underlying ASD, and she agreed for a CARS2-HF assessment. She scored 30 on CARS2-HF, signifying mild to moderate symptoms of autism spectrum disorder. After the psychoeducation session, she requested more material to read about high-functioning autism. She returned to the clinic after a week and reported feeling relieved and accepted her clinical diagnosis. She agreed to pursue weekly supportive psychotherapy. During her subsequent one-month follow-up visit, the patient reported subjective improvement in the affective symptoms with therapy.

Case III

Patient is a 24-year-old female student who aspired to work for animals. She was referred to a psychiatric clinic by her friend. She complained of mood swings and difficulty maintaining a routine. She had loss of appetite, increased need for sleep, and lack of motivation. She reported that she was missing her office and eventually quit her job. Her work was exhausting, and on weekends she slept for 12-14 hours to catch up. During college years, she also struggled with attending classes, and maintaining relationships was stressful. She felt that her personal space was taken over, and therefore she broke up with her boyfriend. During assessment she reported narrow interest in animals; she described different animals in a pedantic manner. She has been interested in animals since early childhood. She had no friends in childhood and interacted mainly with her older brothers’ friends. She struggled with sustaining friendships and did not interact with people much. She shared that she felt different from others and in a large group often struggled to interact with others. During assessment she reported that she imitates others’ accent, expressions, and way of talking to fit in during social interactions. She often ends up upsetting others as she comes across as too honest. She enjoyed spending time with animals more than meeting friends and prefers detailed instructions to complete a task. She had an aversion for tight jeans, wool sweaters, and wet socks. She consulted a psychologist once in the past and had no other relevant medical history. She scored 29.5 on CARS2-HF, signifying mild to moderate symptoms of autism spectrum disorder. She was receptive to the diagnosis of ASD and during psychoeducation sessions asked many questions pertinent to the presentation and diagnosis. She was recommended weekly psychotherapy sessions. During her one-month tele-psychiatry follow-up, she reported to have made clinical improvement with ongoing therapy and reading self-help books.

Case IV

Patient is a 20-year-old female college student. She was referred by her psychologist for mood swings, irritability, and anxiety. She struggled in maintaining relationships, had limited friends, and most of them were on social media. During assessment she reported preference for a wide circle of acquaintances and social media followers. She had a poor relationship with her parents and blamed them for her problems. She felt her brother was closer to her parents, and she often felt neglected. She struggled to maintain a daily routine, often skipped her meals, and took bath on alternative days. She liked socializing but lacked social pragmatics and was often perceived as rude which affected her relationships. She had previously been treated for anxiety by her primary care physician with low-dose clonazepam without any significant benefits. She also had a history of migraines. During assessment she was socially different, had excellent vocabulary with good command over English language with a monotonous speech, and minimal prosody. She reported that she had few friends during her childhood and was enrolled in a special curriculum for gifted kids. She had a gift of remembering dates and would memorize topics in geography. She had a history of sexual abuse at age 18 by a work colleague and reported flashbacks, hypervigilance, and anxiety. She felt that she had borderline personality disorder (BPD), and she could relate to some BPD symptoms such as unstable self-image, feelings of emptiness, and intense interpersonal relationships. She reported sensory symptoms like sensitivity to loud noise and rough texture of clothing. She scored 33.5 on CARS2-HF, signifying mild to moderate symptoms of autism spectrum disorder. In her second meeting with the psychiatrist, she reported that after reading about Asperger’s syndrome, she could relate to it more than BPD. She was prescribed escitalopram 10 mg once daily for her anxiety and trauma symptoms and also recommended trauma therapy once a week. She was seen on the next follow-up visit with her parents, who were also provided psychoeducation about her diagnosis and treatment. She was not able to tolerate an increase in the escitalopram to 15 mg due to sedation, and it was lowered back to 10 mg. The patient after three months of treatment demonstrated good response with the interventions. The patient also reported improvement in her relationship with the family.

## Discussion

Late diagnosis of ASD has serious implications on understanding and interventions for impairments related to these disorders [9-10]. In clinical practice, the current gold standard measures like Autism Diagnostic Observation Schedule (ADOS), which is used for ASD diagnosis for females have false negative results due to several factors [11]. In the past there has been criticism of DSM V and ICD 10 not being able to truly capture the psychopathology distinct to these individuals [12].There are many population-based studies, which show that females with ASD are diagnosed much later in life [13]. Many who failed to get diagnosed early learn to adapt using unique social coping strategies. There are currently no gender-specific validated instruments that could be used for screening these individuals. These attempts to fit in, compensate, and masking behaviors are termed as camouflaging. Recent literature describes these behaviors also include making eye contact, following social script, and mimicking others’ social behaviors [14]. Females use more gestures as compared to males, which is more detailed description of how camouflaging leads to missing a diagnosis [15]. Girls used more “um” as compared to “uh,” which is more sophisticated linguistic camouflaging [16]. These words are pragmatically different when used to fill gaps during pauses and are seen to be used more by ASD girls as compared to boys. Since camouflaging is cognitively exhausting, it often leads to heightened stress response, anxiety, and depression [17]. It is our hypothesis that at this point, most of these patients seek help. In clinical settings, most of these girls presented with affective and anxiety symptoms.

Besides camouflaging, there are other symptoms that are important and distinct to these patients. These patients have all reported that they consider themselves different than other females of their age. They are different in terms of having less need for friends, preference for solitary activities, and lack of initiation to interact socially when in a group. We have also observed less attention to personal appearance, and their dressing does not follow trends and fashion. They also seem to have a clear interest from early teenage life and would often give precise answer about future goals. For example, “I want to be an orthopedic surgeon,” “I want to be a climate scientist,” “I will be a coroner,” “I want to be a youtuber,” etc. They often describe themselves pansexual but have little or no interest in sexual intimacy. They like reading books, are interested in different cultures (e.g., Japanese), like to learn new languages (e.g., Mandarin), and spend a lot of time on video games, in particular Minecraft. Most of them describe themselves as agnostic or atheist when asked about religious affiliation and have little interest in politics. Besides depression and OCD, many patients are often diagnosed with BPD [18], which often leads to hopelessness and self-blame. Based on these findings, these patients may never get accurately diagnosed, and therefore, true estimate of the rate of completed suicide is unknown. In a 2014 study, a group attempted to investigate anecdotal reports of increased suicidality among AS. It found higher suicidal ideation rates among AS even though depression rates were low [19]. It could be due to underreporting of their symptoms due to alexithymia. In clinical samples, rates are higher due to late diagnosis (median age of diagnosis is almost 31 years) and exacerbation due to lack of support during adult life.

## Conclusions

The majority of these patients prefer to do research and read literature before they accept the diagnosis. Future research is heading toward developing measures for camouflaging, compensation, and linguistics unique to this subgroup among ASD. There is a need to educate the clinical community about these new developments. The likelihood of other confounding variables that may affect accurate diagnosis and treatment remains high due to complex presentations. The majority of these patients present to adult psychiatrists who have no formal training to diagnose these individuals. It is therefore of high importance to educate with emerging research to identify these individuals. With appropriate diagnosis, these patients often demonstrate clinical improvement. They feel more accepted and develop therapeutic alliance with clinician. There is a need for more research and recognition of this condition.
